# A Study of Subliminal Emotion Classification Based on Entropy Features

**DOI:** 10.3389/fpsyg.2022.781448

**Published:** 2022-03-25

**Authors:** Yanjing Shi, Xiangwei Zheng, Min Zhang, Xiaoyan Yan, Tiantian Li, Xiaomei Yu

**Affiliations:** ^1^School of Information Science and Engineering, Shandong Normal University, Jinan, China; ^2^Network Information Center, Shandong University of Political Science and Law, Jinan, China; ^3^Geriatrics Center, Affiliated Hospital of Shandong University of Traditional Chinese Medicine, Jinan, China; ^4^Faculty of Education, Shandong Normal University, Jinan, China

**Keywords:** EEG, subliminal emotion, feature extraction, subliminal emotion classification, improved random forest

## Abstract

Electroencephalogram (EEG) has been widely utilized in emotion recognition. Psychologists have found that emotions can be divided into conscious emotion and unconscious emotion. In this article, we explore to classify subliminal emotions (happiness and anger) with EEG signals elicited by subliminal face stimulation, that is to select appropriate features to classify subliminal emotions. First, multi-scale sample entropy (MSpEn), wavelet packet energy (*E*_*i*_), and wavelet packet entropy (WpEn) of EEG signals are extracted. Then, these features are fed into the decision tree and improved random forest, respectively. The classification accuracy with *E*_*i*_ and WpEn is higher than MSpEn, which shows that *E*_*i*_ and WpEn can be used as effective features to classify subliminal emotions. We compared the classification results of different features combined with the decision tree algorithm and the improved random forest algorithm. The experimental results indicate that the improved random forest algorithm attains the best classification accuracy for subliminal emotions. Finally, subliminal emotions and physiological proof of subliminal affective priming effect are discussed.

## 1. Introduction

Affective computing is a multidisciplinary field involving computer science, psychology, and cognitive science and its potential applications include disease diagnosis, human-computer interaction (HCI), entertainment, autonomous driving assistance, marketing, teaching, etc., (Bota et al., 2019). The intelligent brain-computer interface (BCI) systems based on electroencephalogram (EEG) can promote the continuous monitoring of fluctuations in the human brain area under the emotional stimulation, which is of great significance for the development of brain emotional mechanisms and artificial intelligence for medical diagnosis (Gu et al., 2021).

Emotion research also has important findings for neurological-marketing that local neuronal complexity is mostly sensitive to the affective valance rating, while regional neuro-cortical connectivity levels are mostly sensitive to the affective arousal ratings (Aydın, 2019). There is an attention bias processing mechanism for emotions. Some studies have shown that angry faces can automatically stimulate attention, that is, there is an anger dominance effect. On the contrary, some studies have shown the existence of a happiness dominance effect (Xu et al., 2019). Most psychologists regard subjective experiences as the central component of emotion, emphasizing the role of consciousness in emotional production and emotional state. However, the discussion of emotional issues from the perspective of unconscious emotion also has a profound tradition in psychological research. Unconscious emotion, also named subliminal emotion, refers to the change of thoughts and emotions caused by certain emotional states (Li and Lv, 2014). This emotional state is independent of his conscious awareness, and the induction of this emotional state is unconscious (Jiang and Zhou, 2004; Wataru et al., 2014; Zheng et al., 2021a). The presentation of stimuli subliminally is an important research topic in the field of unconscious perception.

Researchers use subliminal stimulus to trigger unconscious states to analyze changes in mood, cognition, social information processing, and physiological signals. Emotional faces are an important and unique visual stimulus and humans are very sensitive to emotional faces and have complex and efficient recognition ability of them. Subliminal emotional face experiments use emotional faces as stimulus materials for subliminal presentation, and they will trigger unconscious emotion (Yin et al., 2021).

We have con'ducted a study on multi-scale sample entropy (MSpEn) (Shi et al., 2018) and in this article, we study new features which are also suitable for the subliminal emotion classification based on EEG signals. These features including MSpEn, WpEn, and *E*_*i*_ extracted from EEG signals have been employed as input feature vectors for classification of subliminal emotions. We combine them with decision tree algorithm and improved random forest to classify subliminal emotions.

This article is structured as follows: Section 2 introduces related work. Section 3 presents the experimental process, subjects, the feature extraction method, and classification algorithms. Section 4 describes the experiments and results. Finally, section 5 concludes this article.

## 2. Related Work

The methods based on physiological signals are more effective and reliable because humans can not control them intentionally, such as electroencephalogram, electromyogram (EMG), electrocardiogram (ECG), skin resistance (SC) (Kim et al., 2004; Kim and Andr, 2008), pulse rate, and respiration signals. Among these methods, EEG-based emotion recognition has become quite common in recent years. There are many research projects focusing on EEG-based emotion recognition (Hosseini and Naghibi-Sistani, 2011; Colic et al., 2015; Bhatti et al., 2016). Jatupaiboon et al. (2013) indicated that the power spectrum from each frequency band is used as features and the accuracy rate of the SVM classifier is about 85.41%. Bajaj and Pachori (2014) proposed new features based on multiwavelet transform for the classification of human emotions from EEG signals. Duan et al. (2013) proposed a new effective EEG feature named differential entropy to represent the characteristics associated with emotional states.

Extracting effective features is the key to the subliminal emotion recognition of EEG signals. Four different features (time domain, frequency domain, time-frequency based, and non-linear) are commonly identified in the feature extraction phase. Compared to traditional time domain and frequency domain analysis, time-frequency based, and non-linear are more widely used (Vijith et al., 2017). Wavelet packet transform is a typical linear time-frequency analysis method. Wavelet packet decomposition is a wavelet transform that provides a time-frequency decomposition of multi-level signals. Murugappan et al. (2008) used video stimuli to trigger emotional responses and extract wavelet coefficients to obtain the energy of the frequency band as input features. Verma and Tiwary (2014) used discrete wavelet transform for feature extraction and classified emotions with support vector machine (SVM), multilayer perceptron (MLP), K nearest neighbor, and metamulticlass (MMC). In recent years, many scholars have tried to analyze EEG signals by non-linear dynamics methods. Commonly used methods are correlation dimension, Lyapunov exponent, Hurst exponent, and other entropy-based analysis methods (Sen et al., 2014). Hosseini and Naghibi-Sistani (2011) proposed an emotion recognition system using EEG signals, and a new approach to emotion state analysis by approximate entropy (ApEn) and wavelet entropy (WE) is integrated. Xin et al. (2015) proposed an improved multi-scale entropy algorithm for emotion EEG features extraction. Michalopoulos and Bourbakis (2017) applied multi-scale entropy (MSE) to EEG recordings of subjects who were watching musical videos selected to elicit specific emotions and found that MSE is able to discover significant differences in the temporal organization of the EEG during events that elicit emotions with low/high valence and arousal.

The upsurge in the study of emotion research attracts scholars to explore and discover subliminal emotions. The analysis and processing of EEG signals have become an indispensable research focus in emotion recognition.

## 3. EEG Data Acquisition and Analysis Methods

**Figure 1:**
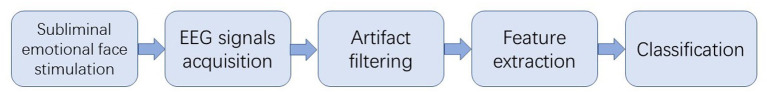
The process of subliminal emotion classification.

The process of subliminal emotion classification consists of several steps as shown in Figure 1. First, a stimulus such as picture, audio, or movie is needed. During the experiments, the participant is exposed to the stimuli to elicit emotion, and EEG signals are recorded accordingly. In order to trigger subliminal emotion, we set the presentation time as 33 ms. Then, artifacts that contaminate EEG signals are removed. EEG signals are analyzed and relevant features are extracted. Some data are used to train the classification model, and the remainder is used for the test which is classified using this model to compute accuracy (Zheng et al., 2021b). Age and gender specifications of the subjects would be given in the Supplementary Material.

### 3.1. Method

#### 3.1.1. Feature Extraction

This article mainly adopts three features, including MSpEn, wavelet packet energy (Ei) and wavelet packet entropy (WpEn). MSpEn is a combination of sample entropy and multi-scale analysis (Klauer and Musch, 2003; Bai et al., 2007). The calculation steps of MSpEn are described in detail in Shi et al. (2018).

##### 3.1.1.1. Wavelet Packet Energy

Wavelet packet decomposition is a generalization of the wavelet transform, which is with multi-resolution characteristics. It can finely analyze signals more than wavelet analysis, so it is very suitable for processing non-stationary signals such as EEG signals and has been widely used in the field of EEG signal processing. Wavelet transform is a multi-scale signal analysis method. It can characterize local features of signals in both time and scale (Deng et al., 2011). The continuous wavelet transform of the signal *f*(*t*) is defined as


(1) $$\begin{array}{l} W_{x}\left(a,b\right)=\frac{1}{\sqrt{a}}\int f\left(t\right)\psi \left(\frac{t-b}{a}\right)dt \end{array}$$

where *a* is the scaling parameter, *b* is the translation parameter, ψ(*t*) is the wavelet function, and *t* is the time.

The discrete wavelet transform is defined as


(2) $$\begin{array}{l} C_{j,k}=\int_{-\infty }^{+\infty }f\left(t\right)\psi_{j,k}\left(t\right)dt \end{array}$$

where $\psi_{j,k}\left(t\right)=2^{-\frac{J}{2}}\psi \left(2^{-j}t-k\right)$.

Wavelet analysis has been widely used in various fields as the main tool for time-frequency analysis. Compared to Fourier transform and short-time Fourier transform, wavelet analysis has the advantage of multi-resolution analysis, which can reflect the local details of signals on multiple scales. However, the traditional wavelet transform only further decomposes the low-frequency components of each decomposed signal. The high frequency components are ignored and the signal details are not adequately reflected. Wavelet packet decomposition is a generalization of the wavelet transform, which is with multi-resolution characteristics. In order to extract the EEG features, the original signal is decomposed by the Mallat algorithm, and the wavelet coefficients of the corresponding nodes are reconstructed to obtain the final coefficients.

To reduce noise and other factors, high frequency components are filtered out, leaving a frequency range below 256 Hz. After four layers wavelet decomposition, the original signal can be decomposed into 16 bands.

The wavelet packet energy of band *i* (*E*_*i*_) is defined as


(3) $$\begin{array}{l} E_{i}=\sum_{i=1}^{N}||n_{j}{||}^{2} \end{array}$$

where *N* is the number of corresponding band coefficients, *n*_*i*_ is the wavelet packet coefficient. The total wavelet packet energy is defined as


(4) $$\begin{array}{l} E_{total}=\sum_{i=1}^{2^{i}}E_{i} \end{array}$$

The wavelet packet energy distribution is expressed as


(5) $$\begin{array}{l} P_{i}=\frac{E_{i}}{E_{total}} \end{array}$$

**Figure 2:**
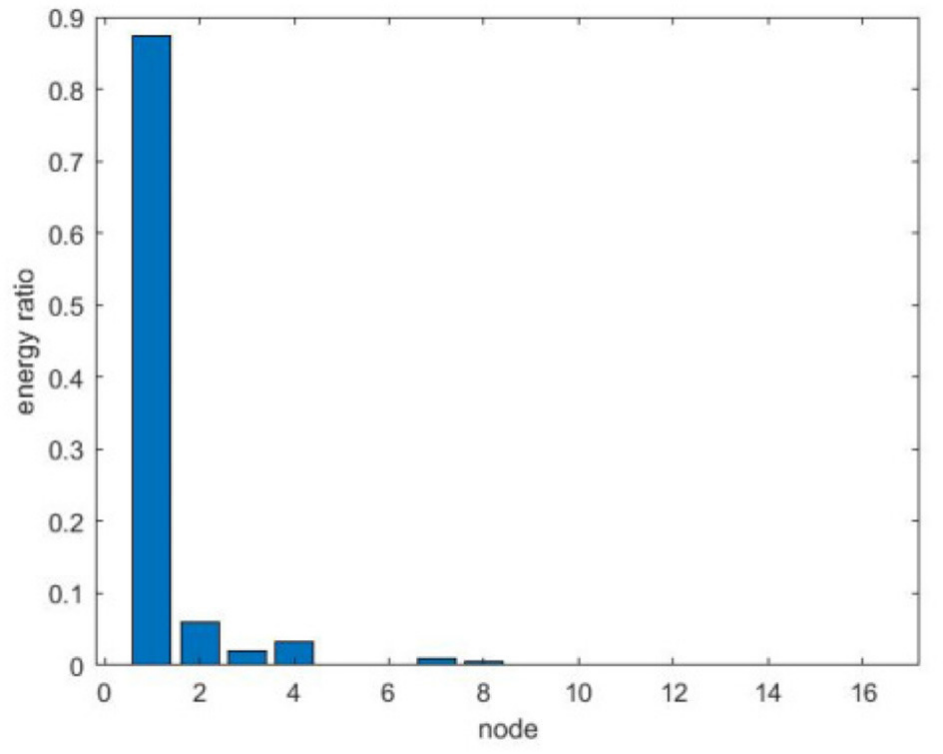
Energy ratio of 4-layer wavelet packet decomposition.

The energy ratio of each wavelet packet node is calculated after the wavelet packet decomposition of the original EEG signals. The result is shown in Figure 2. The energy is concentrated in the frequency band corresponding to the first four wavelet packet nodes after the wavelet packet is decomposed. The energy ratio and corresponding frequency range of the first four wavelet packet nodes are shown in Table 1. More than 98% of the energy is concentrated in the wavelet packet nodes (4,0) ~ (4,3), this is because the human EEG sub-band intervals are as follows: delta, 0.5–4 Hz; theta, 4.5–8 Hz; alpha, 8.5–16 Hz; beta, 16.5–32 Hz; gamma, 32.5–60 Hz. According to the EEG rhythm theory, it means that we only need to extract some features that can cover the human brain frequency range.

**Table 1:** The 4-layer wavelet packet decomposition frequency intervals and energy ratio.

**Wavelet packet node**	**Wavelet packet energy distribution**	**Frequency interval (Hz)**
(4,0)	87.3802%	0 ~ 16
(4,1)	5.9086%	16 ~ 32
(4,2)	3.1553%	32 ~ 48
(4,3)	2.004%	48 ~ 64

According to the above analysis, EEG activities are mainly concentrated in the (4,0) ~ (4,3). Therefore, it is not necessary to use all frequency bands in actual analysis. In order to cover the EEG rhythm as much as possible and avoid the effects of noise and artifacts in EEG records, this article only deals with wavelet packet nodes. The wavelet packet energy of the packet node (4,0) ~ (4,3) is extracted and analyzed whether it is a contribution to subliminal emotion face recognition.

##### 3.1.1.2. Wavelet Packet Entropy

Information entropy can provide a quantitative measure of information contained in various probability distributions. It is a measure of the degree of uncertainty and can be used to estimate the complexity of random signals. The energy distribution of wavelet packet decomposition coefficient and information entropy are combined to define WpEn as:


(6) $$\begin{array}{l} \text{WpEn}=-\sum P_{i}\ln\;P_{i} \end{array}$$

#### 3.1.2. Classification Algorithm

This study employed and evaluated two classifiers, the decision tree algorithm (Yang and XU, 2017) and the improved random forest algorithm (Paul et al., 2018), for subliminal emotion classification. This study systematically compared the effects of all the feature types (MSpEn, WpEn, and *E*_*i*_) on the classification performance.

##### 3.1.2.1. Decision Tree Algorithm

Classification is one of the most widely studied and applied methods in the field of data mining. The decision tree algorithm is widely used because of its fast classification, high precision, and easy-to-understand classification rules. The popular algorithms in the decision tree algorithm are ID3, C4.5, CART, and CHAID. ID3 algorithm based on information entropy is a classic algorithm of decision tree algorithm. The possibility of attribute splitting will increase as the information gain increases. However, ID3 can only deal with discrete properties, while the C4.5 algorithm can handle both discrete and continuous properties. C4.5 algorithm is one of the most widely studied algorithms in decision tree algorithms and is also one of the representative algorithms of decision trees.

The C4.5 algorithm is an improved algorithm of the ID3 algorithm. It uses the information gain rate to select attributes and prunes during tree construction. It can process both discrete and continuous attributes, as well as default data.

The core idea of the C4.5 decision tree algorithm is to use the principle of information entropy to select the attribute with the largest information gain rate as the classification attribute, recursively construct the branches of the decision tree, and complete the construction of the decision tree.

C4.5 algorithm can be described in the following steps:

Step 1: The training data set is preprocessed. If there are continuous attributes in the data set, it needs to be discretized first.
Step 2: The data is classified according to the respective attributes of the data set, and the information gain rate is calculated for each classification result.

Set the training set as *D* and |*D*| indicates the number of records of *D*. The label set of class *D* is *C*, *C* = {*C*_1_, *C*_2_, ..., *C*_*m*_}, where |*C*_*i*_| represents the number of records of *C*. The training set can be divided into *m* different subsets *D*_*i*_, (1 ≤ *i* ≤ *m*) according to labels. Set the attributes set of D as *A*_*n*_, where *A*_*n*_ = {*A*_1_, *A*_2_, ..., *A*_*n*_}, the *i*th attribute of *A*_*i*_ with *w* different values is defined as {*a*_1*i*_, *a*_2*i*_, ..., *a*_*wi*_}. The data set is divided into w different subsets $D_{i}^{A}$, (1 ≤ *i* ≤ *w*) according to the attribute, where $\left|D_{i}^{A}\right|$ represents the number of samples in the subset $D_{i}^{A},|C_{i}^{A}|$ represents the number of *C*_*i*_ in the subset $D_{i}^{A}$. So, the information entropy is defined as


(7) $$\begin{array}{l} Entropy\left(D\right)=-\sum_{i=1}^{m}p_{i}lb\left(p_{i}\right) \end{array}$$

where *p*_*i*_ = |*C*_*i*_|/|*D*|. The information entropy of subset divided according to attribute *A*_*i*_ is defined as


(8) $$\begin{array}{l} EntropyA\left(D\right)=-\sum_{i=1}^{w}\frac{\left|D_{i}^{A}\right|}{\left|D\right|}Entropy\left(D_{i}^{A}\right) \end{array}$$

where $Entropy\left(D_{i}^{A}\right)$ is the information entropy of subset $D_{i}^{A}$. The formula *Gain*(*A*_*i*_) represents the information gain of the training set divided by the attribute *A*_*i*_, which is defined as


(9) $$\begin{array}{l} Gain\left(A_{i}\right)=Entropy\left(D\right)-EntropyA\left(D\right) \end{array}$$

The split information *SplitInfoA*(*D*) is defined as


(10) $$\begin{array}{l} SplitInfoA\left(D\right)=-\sum_{i=1}^{w}\frac{D_{i}^{A}}{D}lb\frac{D_{i}^{A}}{D} \end{array}$$

The information gain ratio of the dataset divided by the attribute *A*_*i*_ is defined as


(11) $$\begin{array}{l} GainRatio\left(A_{i}\right)=\frac{Gain\left(A_{i}\right)}{SplitInfoA\left(D\right)} \end{array}$$

Step 3: According to the attribute which is corresponding to the maximum information gain ratio, the current data set is divided into different subsets, the decision tree branches are established, and the new child nodes are formed.
Step 4: Steps 2 and 3 are recursively called for the new node until the class labels are the same in all nodes.

##### 3.1.2.2. Improved Random Forest Algorithm

Random forest algorithm is a typical multi-classifier algorithm. The basic classifier that constitutes the random forest algorithm is the decision tree. The basic principle of the random forest algorithm is to use the resampling technique to form a new training set by randomly extracting samples. Then, decision tree is modeled and composed of random forests, and the classification results are used for voting decisions (Bo, 2017).

The random forest algorithm is similar to the Bagging algorithm in that it is resampled based on the bootstrap method to generate multiple training sets. The difference is that the random forest algorithm uses the method of randomly selecting the split attribute set when constructing a decision tree.

Random forest algorithm can be divided into the following steps:

Step 1: Use the bootstrap method to resample and randomly generate *T* training sets, *S*_1_, *S*_2_, , *S*_*T*_.
Step 2: Generate a corresponding decision tree using each training set; before selecting attributes on each internal node, *m* attributes are randomly extracted from *M* attributes as the split attribute set of the current node, and the node is split by the best classification among the *m* attributes.
Step 3: Every tree grows intact without pruning.
Step 4: For the test set sample *X*, use each decision tree to test and get the corresponding category.
Step 5: Using the voting method, the category with the most output in the *T* decision trees is taken as the category to which the test set sample *X* belongs.

However, the random forest algorithm also has deficiencies. This article uses a random forest algorithm based on the C4.5 tree algorithm. The attribute division strategy is based on the level of information gain rate to select the characteristics of the division. The principle of attribute division has the disadvantage of biasing features with many values. The voting on the classification result of the decision tree adopts the “majority voting principle,” which means the number of votes is the final classification result, and the strength of the decision tree classifier cannot be distinguished. This article will improve the random forest algorithm and apply it to the classification of subliminal emotional faces.

Random forest algorithm is improved from the following mechanisms:

(1) In the choice of test attributes, attribute division by information gain rate will have the characteristic of biasing the features with more values, the Pearson coefficient is introduced to compensate.

The C4.5 decision tree algorithm uses the information gain ratio to select the test attribute. The larger the information gain ratio, the stronger the correlation between the attribute and the class attribute, the possibility of the attribute being selected as the test attribute the larger.

The C4.5 decision tree algorithm takes into account the influence of attributes on class, but it does not involve the influence between attributes. If an attribute has a strong correlation with other attributes, there will exist redundancy between them. Therefore, the Pearson coefficient is used to express the temporal correlation of attributes in this article, and the influence of attributes with high correlation is eliminated. The quotient of the covariance and the standard deviation is defined as the Pearson correlation coefficient, which can reflect the degree of correlation between two variables. The Pearson coefficient is defined as


(12) $$\begin{array}{l} r=\frac{\sum XY-\frac{\sum X\sum Y}{N}}{\sqrt{\left(\sum X^{2}-\frac{{\left(\sum X\right)}^{2}}{N}\right)\left(\sum Y^{2}-\frac{{\left(\sum Y\right)}^{2}}{N}\right)}} \end{array}$$

When the Pearson coefficient is 0, it means there is no correlation between two variables. When the Pearson coefficient is positive, it indicates that there is a positive correlation between the two variables. The larger the value, the greater the positive correlation between the two variables. When the Pearson coefficient is negative, it means that there is a negative correlation between two variables. The greater the value, the greater the negative correlation between two variables. The range of Pearson's coefficient is (−1, 1).

The improved attribute division algorithm is defined as


(13) $$\begin{array}{l} GainRatio\left(A_{i}\right)=\frac{Gain\left(A_{i}\right)}{SplitInfoA\left(D\right)+ar} \end{array}$$

According to the improved attribute selection method, the attribute which has the high information gain ratio and low correlation with other attributes, has the greater probability of being selected as the test attribute.

(2) The voting decision process is an important mechanism of the random forest algorithm. Random forest algorithm adopts the principle of majority voting, assigning each decision tree the same weight, and ignoring the difference between the strong classifier and the weak classifier, which affects the overall classification performance of random forest. This article uses the weighted voting principle to improve random forest algorithm. During the formation of the random forest, according to the classification performance of each decision tree, each decision tree is assigned a corresponding weight. Then, the final classification effect is obtained by weighted voting.

**Figure 3:**
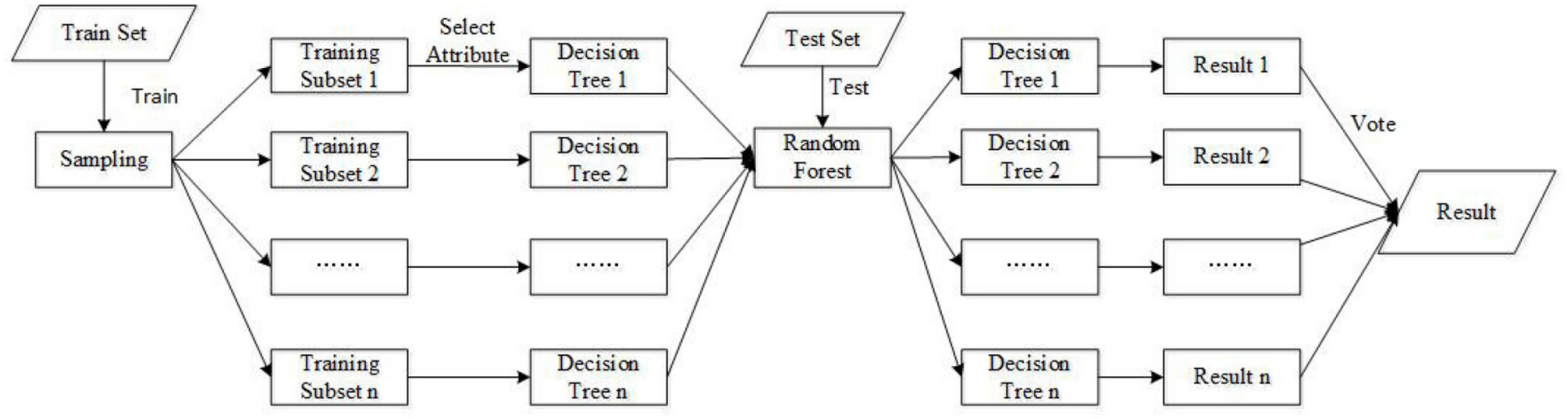
The principle of improved random forest algorithm.

In the process of generating a decision tree, the bagging method is used to extract samples from original training set with replacement to form a sample set, and the decision tree classification accuracy rate *Ac*_*t*_*ree* corresponding to the sample set can be obtained. The larger *Ac*_*t*_*ree*, the better the classification effect of the decision tree, which belongs to the strong classifier. *Ac*_*t*_*ree* of each decision tree is used as the weight of the corresponding decision tree and add the weights corresponding to decision trees with the same output class target. Finally, classification result with higher weight is the final category. Figure 3 shows the schematic diagram of the improved random forest algorithm.

## 4. Results

First, low pass filtering is applied to each EEG signal segment. According to the sampling theorem, the maximum frequency of the signal is about 500 Hz. To reduce noise and other factors, high frequency components are filtered out, leaving a frequency range below 256 Hz. The sample entropy has a strong ability to characterize nonlinear sequences on a macroscopic scale and cannot describe the details. The wavelet packet decomposition has excellent description ability in detail. Therefore, MSpEn, WpEn, and *E*_*i*_are extracted as feature vectors for the classifier, and the classification results of different features are compared. Bai et al. (2007) pointed out that when the sample entropy parameter *m* = 2 and *r* = 0.2*STD* are selected through experiments, the classification effect is better. In addition, Duan et al. (2013) pointed out that the scale factor *t* = 2 is preferentially chosen. The wavelet basis function selects the db-4 wavelet. We can get 16 bands after the four-layer wavelet packet decomposition of the signals and we calculate the energy ratio of each node after wavelet packet decomposition according to the formula (5).

In our experiment, there were 80 sets of sample data for each subject, 40 groups were selected as training samples for training the proposed model, and the remaining 40 groups were used as test samples for verifying the performance of the model. WpEn and *E*_*i*_ extracted by wavelet packet transform and MspEn calculated by multi-scale sample analysis is put into decision tree algorithm, respectively. The averaged classification performance of the decision tree algorithm with MSpEn, WpEn, and *E*_*i*_ on 10 subjects is shown in Table 2.

**Table 2:** Average results of decision tree algorithm with multi-scale sample entropy (MSpEn), wavelet packet entropy (WpEn), and wavelet packet energy (*E*_*i*_).

**Method**	**Subject 1**	**Subject 2**	**Subject 3**	**Subject 4**	**Subject 5**	**Subject 6**
MSpEn	80.25%	73.57%	72.15%	53.65%	83.52%	79.80%
*E*_*i*_	**97.75%**	**85.02%**	96.77%	**96.05%**	**96.75%**	**91.87%**
WpEn	96.97%	83.27%	**97.60%**	95.30%	91.40%	88.68%
**Method**	**Subject 6**	**Subject 7**	**Subject 8**	**Subject 9**	**Subject 10**	**Average**
MSpEn	79.80%	75.80%	78.60%	84.50%	73.40%	75.52%
*E*_*i*_	**91.87%**	89.07%	**94.82%**	**97.62%**	**97.57%**	**94.33%**
WpEn	88.68%	**91%**	94.08%	97.55%	97.39%	93.32%

Table 2 shows classification accuracy when MSpEn, *E*_*i*_, and WpEn are input to the decision tree classifier. The experimental results show that decision tree algorithm can effectively classify subliminal emotional faces combined with the three feature vectors, and different feature vectors have different classification capabilities for subliminal emotional faces. The classification accuracy with *E*_*i*_ as a feature vector is significantly higher than other features, and its average classification accuracy is up to 94.33%. The classification accuracy using WpEn as the feature vector is slightly lower, and its average classification accuracy is 93.32%. The classification accuracy with MSpEn as the feature vector is the lowest, and its average classification accuracy is 75.52%.

**Figure 4:**
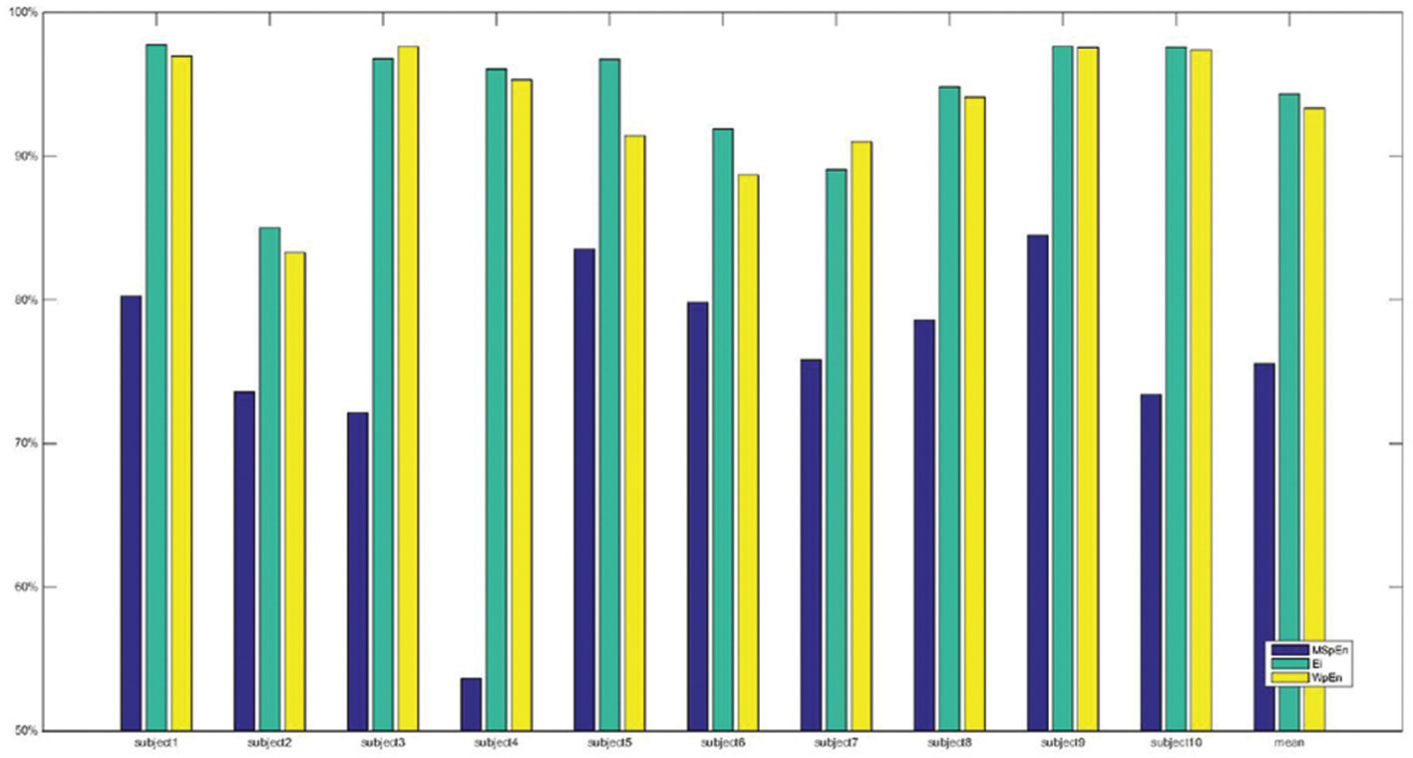
Comparison of classification accuracy with three features and decision tree classifier.

In order to compare the classification performance of different features more intuitively, Figure 4 shows the comparison of the classification accuracy of different features input to the decision tree. It can be seen from Figure 4 that the classification accuracy using MSpEn as the feature vector is the lowest and the accuracy obtained by MSpEn fluctuates greatly in different subjects. When *E*_*i*_ and WpEn are adopted as the input feature vector of the decision tree classifier, the average classification accuracy is significantly higher than MSpEn and the classification accuracy is more stable.

In summary, a decision tree classifier can effectively classify subliminal emotional faces. In the perspective of feature vectors, classification effect of *E*_*i*_ and WpEn is better compared with MSpEn, which shows that wavelet packet decomposition features are more powerful for subliminal emotional face recognition.

This article further studies the classification effect of improved random forest algorithm on subliminal emotional faces. WpEn and *E*_*i*_ extracted by wavelet packet transform and MspEn calculated by multi-scale sample analysis are input into improved random forest, respectively. The averaged classification performance of the improved random forest algorithm with MSpEn, WpEn, and *E*_*i*_ on 10 subjects is shown in Table 3.

**Table 3:** Average results of random forest algorithm with MSpEn, WpEn, and *E*_*i*_.

**Method**	**Subject 1**	**Subject 2**	**Subject 3**	**Subject 4**	**Subject 5**	**Subject 6**
MSpEn	87.65%	85%	86.25%	85%	91.25%	96.25%
*E*_*i*_	**98.75%**	**95%**	**97.5%**	**93.75%**	**98.75%**	**97.5%**
WpEn	96.25%	86.25%	**93.75%**	90%	95%	95%
**Method**	**Subject 6**	**Subject 7**	**Subject 8**	**Subject 9**	**Subject 10**	**Mean**
MSpEn	96.25%	88.75%	86.25%	93.75%	88.75%	88.89%
*E*_*i*_	**97.5%**	96.25%	**95%**	**97.5%**	**97.5%**	**96.75%**
sWpEn	95%	**91%**	91.25%	93.75%	96.25%	93.38%

As we can see from Table 3, three feature extraction algorithms can identify unconscious emotions triggered by subliminal faces stimulus. The classification performance with *E*_*i*_ was evidently better than those based on other feature types under the same conditions, while the accuracy can reach up 96.75%. While WpEn and MSpEn are used as input feature vectors, the unconscious emotions can be classified combined with improved random forest. The highest classification accuracy can be achieved at 93.38 and 88.89%. For the classification results with *E*_*i*_ and the decision tree algorithms and the improved random forest algorithms, it was noted that the random forest algorithms outperformed the decision tree algorithm by 1 ~ 6% for most subjects.

**Figure 5:**
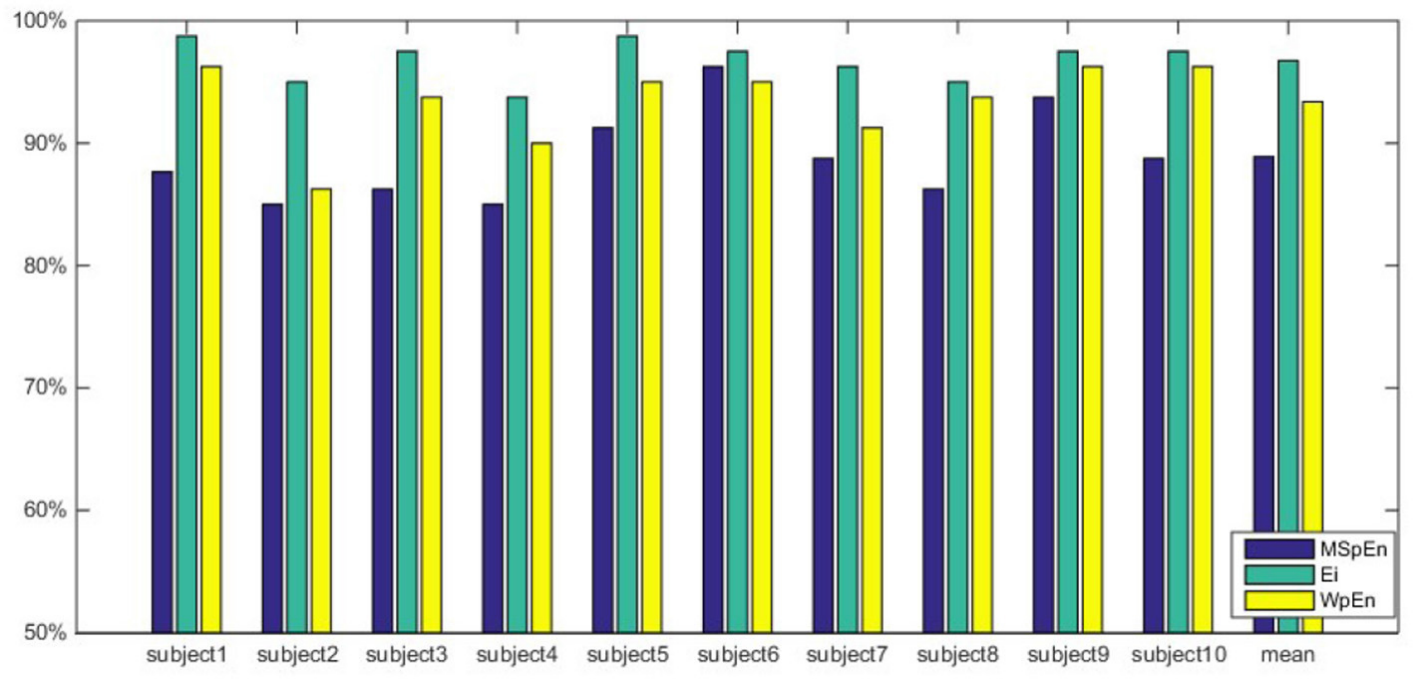
Comparison of classification accuracy with three features and improved fandom forest.

The comparison of classification accuracy of improved random forest classifier with different inputs feature vectors is shown in Figure 5. It can be seen from the figure that the average classification accuracy of a single subject and all subjects are significantly higher when using *E*_*i*_ and WpEn as the input feature vectors of the classifier compared to MSpEn. Due to the individual differences of the subjects, the classification accuracy of different subjects shows a small range of fluctuations, and the overall performance shows that *E*_*i*_ and WpEn have a stronger classification ability than the MSpEn.

**Figure 6:**
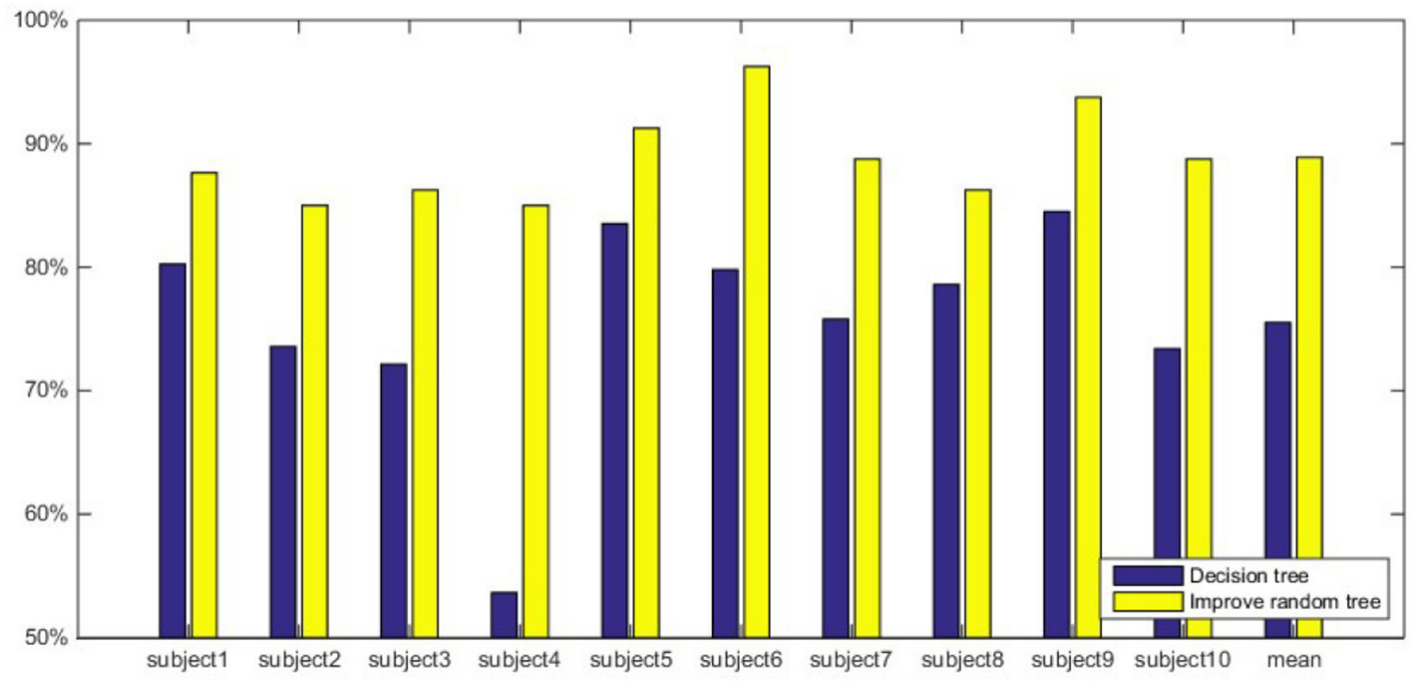
Comparison of classification accuracy of two classifiers based on MSpEn.

**Figure 7:**
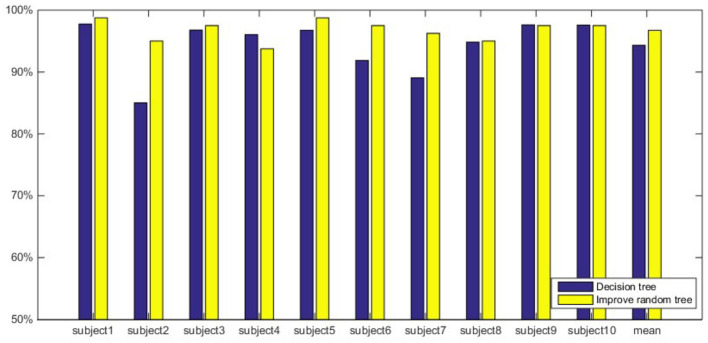
Comparison of classification accuracy of two classifiers based on *E*_*i*_.

**Figure 8:**
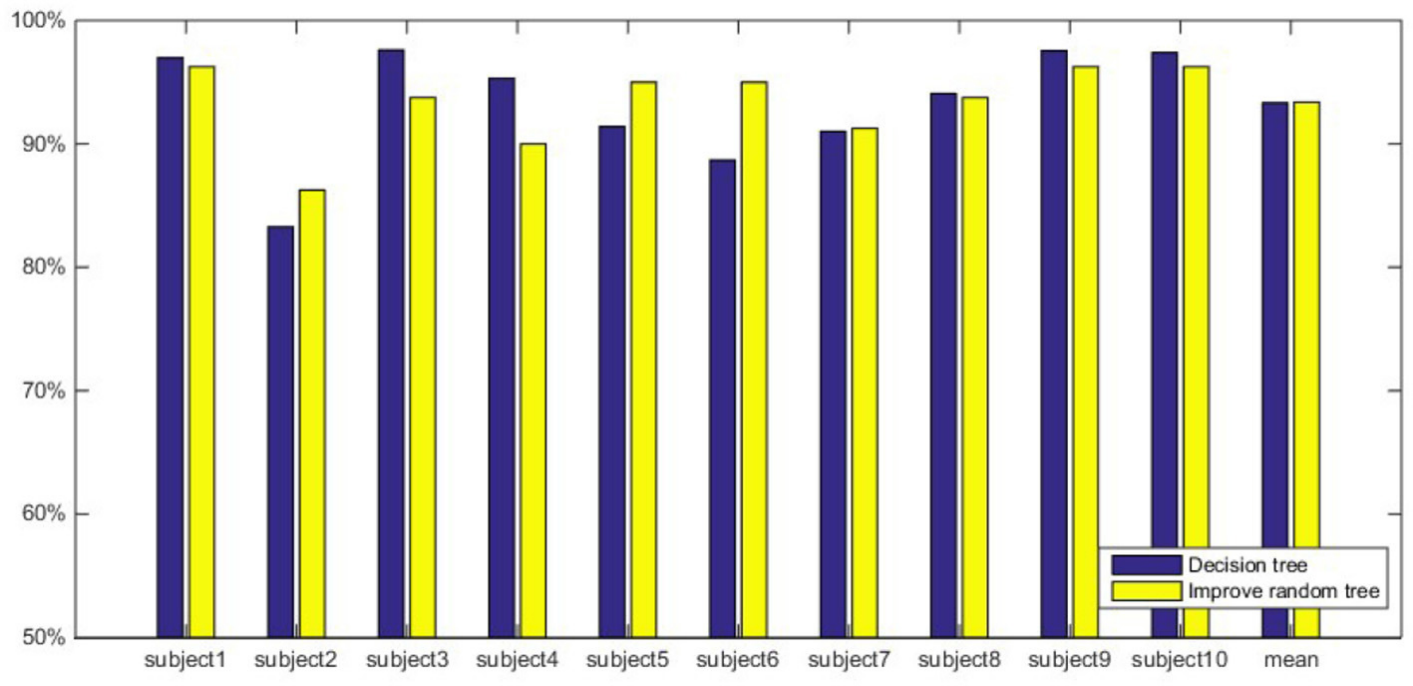
Comparison of classification accuracy of two classifiers based on WpEn.

This article further analyzes and compares the classification accuracy of the two classifiers under the same feature extraction method. The classification results are shown in Figures 6–8. Figure 6 shows the classification results when using MSpEn as an input feature vector. The experimental results show that the improved random forest algorithm shows a stronger classification ability of 10 subjects, and the classification accuracy is significantly higher compared to the decision tree algorithm. Figure 7 shows the classification results when using *E*_*i*_ as input feature vectors. It can be seen that the classification accuracy of the decision tree algorithm of only one subject is higher than that of the improved random forest algorithm. The classification accuracy of the improved random forest algorithm of the remaining 9 subjects is higher than that of the decision tree algorithm. Overall, the classification accuracy of the improved random forest algorithm is higher than that of the decision tree algorithm, and the average classification accuracy is improved by 2.42%. Figure 8 shows the classification results when using WpEn as input feature vectors. When the WpEn is used as the classification feature, the classification accuracy fluctuation between different subjects is more obvious, and the two classification algorithms show different advantages in different subjects. However, from the perspective of average classification accuracy, the classification accuracy of the improved random forest algorithm is slightly higher.

**Figure 9:**
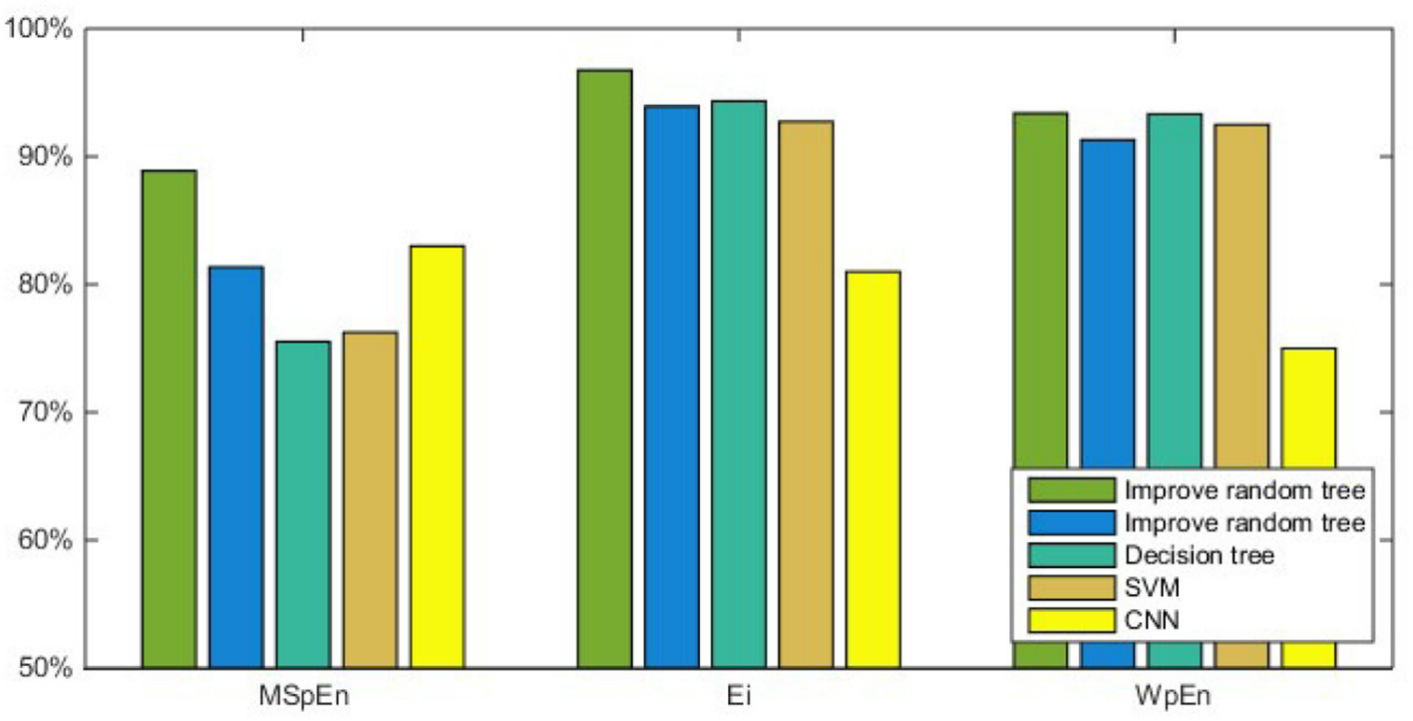
Comparison of the average classification results with other classifiers.

At present, there are few studies and references on subliminal unconscious emotions. In order to confirm the effectiveness of the proposed method, this article compares the classification results of several different classifier algorithms. The experimental results are shown in Figure 9.

In summary, combining three features with decision tree classifier and an improved random forest classifier can realize the classification of subliminal emotional faces. From the perspective of feature extraction, *E*_*i*_ and WpEn obtained by wavelet packet decomposition have obvious advantages for subliminal emotion face classification, and their ability to classify emotional faces is significantly stronger than MSpEn. From the perspective of the classifier, improved random forest is superior to decision tree.

## 5. Conclusion

This article studies features and classification of subliminal unconscious emotions based on EEG signals. We use the subliminal emotional faces as a starting stimulus, in fact, the subjects cannot recognize the emotional content of the face pictures. In the absence of clear emotional information, the human brain can still perform rapid, unconscious processing. We select three effective features first, and then they are combined with a decision tree algorithm and improved random forest algorithms to classify the unconscious emotions triggered by a subliminal stimulus. The experimental results show that classification accuracy of wavelet packet decomposition features (*E*_*i*_ and WpEn) with two classifiers is significantly higher than MSpEn, which shows that wavelet packet decomposition can better characterize the EEG signals triggered by subliminal emotional face stimuli.From the perspective of psychology, we explore the neural mechanisms of brain activity under subliminal face stimulation (Zheng et al., 2021c). Psychological researches show that the presentation of face stimuli at a subliminal time can trigger an emotional priming effect, that is, the initiation of unconscious emotions. Researchers have conducted a lot of experimental investigations on this issue and explored the physiological proof of subliminal emotional priming effects. Some works have shown that the thalamus, hippocampus, amygdala, and their functional connections play an important role in the processing of subliminal emotional faces (Eickhoff et al., 2009). In unconscious situation, humans may have a faster way for processing of emotional faces (especially fearful faces). This way bypasses the primary visual cortex involved in conscious processing, along with the upper mound, the thalamus, and conveys to the amygdala, and then projects to other advanced cortical areas associated with emotional processing (Zhu et al., 2013). Dolan (2002) found that human emotional processing can occur when the subject is unconscious of the process. Smith (2011) work shows that multiple types of emotional faces as fearful faces can be processed at an unconscious level in an early stage.

## Data Availability Statement

The data analyzed in this study is subject to the following licenses/restrictions: The data were collected from college students in our university, while they are not be standardized and opened at present. Requests to access these datasets should be directed to Xiaomei Yu, yxm0708@126.com.

## Ethics Statement

The studies involving human participants were reviewed and approved by Shandong Normal University Ethics Committee. The participants provided written informed consent to participate in this study.

## Author Contributions

XZ designed the project. YS wrote the code. MZ drafted this article. XYa analyzed the data. TL helped analyze the data. XYu revised this article. All authors read and approved this article.

## Funding

This work was supported by the Shandong Provincial Project of Graduate Education Quality Improvement (Nos. SDYJG21104, SDYJG19171, and SDYY18058), the OMO Course Group Advanced Computer Networks of Shandong Normal University, the Teaching Team Project of Shandong Normal University, Teaching Research Project of Shandong Normal University (2018Z29), Provincial Research Project of Education and Teaching (No. 2020JXY012), and the Natural Science Foundation of Shandong Province (Nos. ZR2020LZH008, ZR2021MF118, and ZR2019MF071). The content of this manuscript has been presented in part at the 2018 IEEE International Conference on Bioinformatics and Biomedicine (BIBM) (Shi et al., 2018).

## Conflict of Interest

The authors declare that the research was conducted in the absence of any commercial or financial relationships that could be construed as a potential conflict of interest.

## Publisher's Note

All claims expressed in this article are solely those of the authors and do not necessarily represent those of their affiliated organizations, or those of the publisher, the editors and the reviewers. Any product that may be evaluated in this article, or claim that may be made by its manufacturer, is not guaranteed or endorsed by the publisher.

## References

[B1] Aydın S.. (2019). Deep learning classification of neuro-emotional phase domain complexity levels induced by affective video film clips. IEEE J. Biomed. Health Inf. 24, 1695–1702. 10.1109/JBHI.2019.2959843 31841425

[B2] Bai D. Qiu T. Li X. (2007). The sample entropy and its application in eeg based epilepsy detection. J. Biomed. Eng. 24, 200–205. 10.3321/j.issn:1001-5515.2007.01.043 17333922

[B3] Bajaj V. Pachori R. B. (2014). Human emotion classification from eeg signals using multiwavelet transform, in International Conference on Medical Biometrics (Shenzhen).

[B4] Bhatti A. M. Majid M. Anwar S. M. Khan B. (2016). Human emotion recognition and analysis in response to audio music using brain signals. Comput. Hum. Behav. 65, 267–275. 10.1016/j.chb.2016.08.029

[B5] Bo S.. (2017). Research on the classification of high dimensional imbalanced data based on the optimizational random forest algorithm, in International Conference on Measuring Technology & Mechatronics Automation (New York, NY).

[B6] Bota P. J. Wang C. Fred A. Silva H. P. (2019). A review, current challenges, and future possibilities on emotion recognition using machine learning and physiological signals. IEEE Access 7, 140990–141020. 10.1109/ACCESS.2019.2944001

[B7] Colic S. Wither R. G. Lang M. Liang Z. Bardakjian B. L. (2015). Support vector machines using eeg features of cross-frequency coupling can predict treatment outcome in mecp2-deficient mice, in Engineering in Medicine & Biology Society (Milan). 10.1109/EMBC.2015.7319663 26737563

[B8] Deng W. Miao D. Xie C. (2011). Best basis-based wavelet packet entropy feature extraction and hierarchical eeg classification for epileptic detection. Exp. Syst. Appl. 38, 14314–14320. 10.1016/j.eswa.2011.05.096

[B9] Dolan R. J.. (2002). Emotion, cognition, and behavior. Science 298, 1191–1194. 10.1126/science.1076358 12424363

[B10] Duan R. N. Zhu J. Y. Lu B. L. (2013). Differential entropy feature for eeg-based emotion classification, in Neural Engineering (NER), 2013 6th International IEEE/EMBS Conference on (San Diego, CA).

[B11] Eickhoff S. B. Laird A. R. Grefkes C. Wang L. E. Zilles K. Fox P. T. (2009). Coordinate-based ale meta-analysis of neuroimaging data: a random-effects approach based on empirical estimates of spatial uncertainty. Hum. Brain Map. 30, 2907–2926. 10.1002/hbm.20718 19172646 PMC2872071

[B12] Gu X. Cao Z. Jolfaei A. Xu P. Wu D. Jung T.-P.. (2021). Eeg-based brain-computer interfaces (bcis): a survey of recent studies on signal sensing technologies and computational intelligence approaches and their applications. IEEE/ACM Trans. Comput. Biol. Bioinformat. 18, 1645–1666. 10.1109/TCBB.2021.3052811 33465029

[B13] Hosseini S. A. Naghibi-Sistani M. B. (2011). Emotion recognition method using entropy analysis of eeg signals. Int. J. Image Graph. Signal Process. 3, 30–36. 10.5815/ijigsp.2011.05.05

[B14] Jatupaiboon N. Panngum S. Israsena P. (2013). Emotion classification using minimal EEG channels and frequency bands, in The 2013 10th International Joint Conference on Computer Science and Software Engineering (JCSSE) (Khon Kaen).

[B15] Jiang C. Zhou X. (2004). Emotional automatic processing and control processing. Adv. Psychol. Sci. 12, 688–692. 10.3969/j.issn.1671-3710.2004.05.007

[B16] Kim J. Andr E. (2008). Emotion recognition based on physiological changes in music listening. IEEE Trans. Pattern Anal. Mach. Intell. 30, 2067–2083. 10.1109/TPAMI.2008.26 18988943

[B17] Kim K. H. Bang S. W. Kim S. R. (2004). Emotion recognition system using short-term monitoring of physiological signals. Med. Biol. Eng. Comput. 42, 419–427. 10.1007/BF02344719 15191089

[B18] Klauer K. C. Musch J. (2003). Affective priming: Findings and theories, in The Psychology of Evaluation: Affective Processes in Cognition and Emotion (New Jersey, NJ: Lawrence Erlbaum), 7–50.

[B19] Li T. Lv Y. (2014). The subliminal affective priming effects of faces displaying various levels of arousal: an erp study. Neurosci. Lett. 583, 148–153. 10.1016/j.neulet.2014.09.027 25258346

[B20] Michalopoulos K. Bourbakis N. (2017). Application of multiscale entropy on eeg signals for emotion detection, in IEEE Embs International Conference on Biomedical & Health Informatics (Orlando, FL), 341–344.

[B21] Murugappan M. Rizon M. Nagarajan R. Yaacob S. Zunaidi I. (2008). Time-frequency analysis of EEG signals for human emotion detection, in 4th Kuala Lumpur International Conference on Biomedical Engineering (Kuala Lumpur).

[B22] Paul A. Mukherjee D. P. Das P. Gangopadhyay A. Chintha A. R. Kundu S. (2018). Improved random forest for classification, in IEEE Transactions on Image Processing (IEEE), 4012–4024. 10.1109/TIP.2018.2834830 29993742

[B23] Sen B. Peker M. Cavusoglu A. Celebi F. V. (2014). A comparative study on classification of sleep stage based on eeg signals using feature selection and classification algorithms. J. Med. Syst. 38, 18. 10.1007/s10916-014-0018-0 24609509

[B24] Shi Y. Zheng X. Li T. (2018). Unconscious emotion recognition based on multi-scale sample entropy, in 2018 IEEE International Conference on Bioinformatics and Biomedicine (BIBM) (Madrid: IEEE), 1221–1226.

[B25] Smith M. L.. (2011). Rapid processing of emotional expressions without conscious awareness. Cereb. Cortex 22, 1748–1760. 10.1093/cercor/bhr250 21955918

[B26] Verma G. K. Tiwary U. S. (2014). Multimodal fusion framework: a multiresolution approach for emotion classification and recognition from physiological signals. Neuroimage 102, 162–172. 10.1016/j.neuroimage.2013.11.007 24269801

[B27] Vijith V. S. Jacob J. E. Iype T. Gopakumar K. Yohannan D. G. (2017). Epileptic seizure detection using non linear analysis of eeg, in International Conference on Inventive Computation Technologies (Coimbatore).

[B28] Wataru S. Yasutaka K. Motomi T. (2014). Enhanced subliminal emotional responses to dynamic facial expressions. Front. Psychol. 5, 994. 10.3389/fpsyg.2014.00994 25250001 PMC4158748

[B29] Xin L. Xie J. Hou Y. Wang J. (2015). An improved multiscale entropy algorithm and its performance analysis in extraction of emotion eeg features. Chin. High Technol. Lett. 7, 436–439. 10.1166/jmihi.2017.2031

[B30] Xu Q. He W. Ye C. Luo W. (2019). Attentional bias processing mechanism of emotional faces: anger and happiness superiority effects. Acta Physiologica Sinica 71, 86–94. 10.13294/j.aps.2018.0098 30778507

[B31] Yang H. XU J. (2017). Android malware detection based on improved random forest. J. Commun. 38, 8–16. 10.11959/j.issn.1000-436x.2017073

[B32] Yin Y. Zheng X. Hu B. Zhang Y. Cui X. (2021). Eeg emotion recognition using fusion model of graph convolutional neural networks and lstm. Appl. Soft Comput. 100, 106954. 10.1016/j.asoc.2020.106954

[B33] Zheng X. Liu X. Zhang Y. Cui L. Yu X. (2021a). A portable hci system-oriented eeg feature extraction and channel selection for emotion recognition. Int. J. Intell. Syst. 36, 152176. 10.1002/int.22295

[B34] Zheng X. Yu X. Yin Y. Li T. Yan X. (2021b). Three-dimensional feature maps and convolutional neural network-basedemotion recognition. Int. J. Intell. Syst. 36, 6312–6336. 10.1002/int.2255132240426

[B35] Zheng X. Zhang M. Li T. Ji C. Hu B. (2021c). A novel consciousness emotion recognition method using erp components and mmse. J. Neural Eng. 18, 046001. 10.1088/1741-2552/abea62 33636711

[B36] Zhu X. L. Xiao L. Wen P. (2013). Subliminal emotional face and its brain mechanism. Nat. Defense Sci. Technol. 34, 16–20. 10.3969/j.issn.1671-4547.2013.04.004

