# Supplementary material for: A Study of Subliminal Emotion Classification Based on Entropy Features
Source: Front Psychol. 2022 Mar 25;13:781448. doi: 10.3389/fpsyg.2022.781448 (PMC8989849; doi:10.3389/fpsyg.2022.781448)
Supplement: Supplementary file 1 [file Data_Sheet_1.PDF]

Supplementary Material

1 SUPPLEMENTARY DATA

Age, gender specifications of the participants are given in the Table 1.

Table 1. Age, gender specifications of the participants.

The number of subjects	Age	Gender
**Subject 1**	22	female
**Subject 2**	23	male
**Subject 3**	21	female
**Subject 4**	22	female
**Subject 5**	22	male
**Subject 6**	23	male
**Subject 7**	21	female
**Subject 8**	22	male
**Subject 9**	24	male
**Subject 10**	20	female
**Mean**	22	5 male & 5 female
